# Deformation Prediction of Metro Deep Excavations Using CEEMDAN-IWT Denoising and BO-XGBoost

**DOI:** 10.3390/s26154741

**Published:** 2026-07-26

**Authors:** Jing Zhao, Longhui Chen, Hongyin Yang, Zhuo Hu, Hao Peng, Linlong Yang, Hongyou Cao

**Affiliations:** 1 China Railway 11th Bureau Group First Engineering Co., Ltd., Xiangyang 441199, China; zz871620@163.com (J.Z.); 18244780953@163.com (L.C.); 17380984595@163.com (H.P.); 2 School of Civil Engineering and Architecture, Wuhan Institute of Technology, Wuhan 430073, China; 13125190309@163.com (Z.H.); yanglinlong123@163.com (L.Y.); 3 School of Civil Engineering and Architecture, Wuhan University of Technology, Wuhan 430070, China; caohongyou@whut.edu.cn

**Keywords:** deep excavation, deformation prediction, CEEMDAN-IWT joint denoising, sample entropy adaptive screening, BO-XGBoost model

## Abstract

**Highlights:**

A novel improved wavelet threshold function is proposed for denoising deformation monitoring data, addressing the discontinuity of hard thresholding and the constant bias of soft thresholding.

**What are the main findings?**

- Effective elimination of noise from metro deep excavation monitoring data is achieved by the developed CEEMDAN-IWT joint denoising strategy.
- High-precision prediction of metro deep excavation deformation is achieved using the CEEMDAN-IWT denoised data with a BO-XGBoost model.

**What are the implications of the main findings?**

- Provides a practical solution for denoising and improving the quality of deformation-monitoring data for metro deep excavations.
- Demonstrates a data-driven framework applicable to metro deep excavation deformation prediction.

**Abstract:**

During deep excavation construction, deformation impacts on adjacent structures are inevitably induced, and field monitoring data are often contaminated by noise that degrades prediction accuracy. To address these issues, this study develops a joint denoising strategy combining CEEMDAN, sample entropy-based adaptive IMF screening, and an improved wavelet threshold (IWT) function, followed by a Bayesian optimization-based extreme gradient boosting (BO-XGBoost) model for surface settlement prediction. The developed method automatically identifies high-noise IMF components via sample entropy and processes them using an improved threshold function that overcomes the discontinuity of hard thresholding and the constant bias of soft thresholding, thereby preserving useful information while suppressing noise. Experimental results on a Wuhan metro deep excavation project demonstrate that the CEEMDAN-IWT method improves SNR by up to 4.09% and reduces RMSE by up to 8.00% compared with conventional CEEMDAN-wavelet threshold denoising. The BO-XGBoost model trained on denoised data achieves an RMSE of 0.09 mm and a MAPE of 3.54%, outperforming BP, LSTM, standard XGBoost, GRU, CNN-LSTM, TCN, and simple regression baselines. Feature importance analysis confirms that the denoised data retain physical interpretability consistent with soil deformation continuity. This framework provides a practical solution with promising accuracy for deformation monitoring and early warning in deep excavation engineering.

## 1. Introduction

The rapid growth of urban populations in Chinese cities has intensified pressure on transportation systems, driving continuous expansion in subway construction [1]. To address this, subway construction has been continuously expanded. Deep excavations represent a critical and controlling stage in the development of urban subway networks. The safety of construction activities directly influences the stability of the surrounding environment and the security of public lives and assets. When deep excavations are carried out in dense urban environments, the stress field of the native soil is significantly disturbed, leading to complex deformations in the retaining structures [2]. Therefore, it is essential to control the impact of deep excavation on surrounding buildings and structures [3]. Establishing a high-accuracy predictive model for deep excavation deformation [4] is of paramount importance for achieving advanced risk warning and intelligent construction management. As a key aspect in deep excavation deformation control [5], commonly used prediction methods encompass numerical simulation and machine learning [6,7]. Regarding deep excavation deformation prediction, numerous methods have been proposed by domestic and international scholars for various application scenarios [8,9]. However, prediction models based on a single algorithm [10,11] often exhibit insufficient stability and accuracy. Thus, researchers have developed integrated prediction models by integrating multiple individual models. Experimental results have demonstrated that these integrated models yield superior outcomes compared to single-model approaches [12,13].

Currently, the rapid advancement of sensing technology has enabled high-frequency, real-time monitoring of multiple physical quantities, such as excavation deformation, surface settlement, and horizontal displacement. This capability thereby provides a rich data foundation for data-driven prediction models [14]. In recent years, data-driven computational methods have gained significant attention in geotechnical engineering. A model-free data-driven computational approach was proposed by Zhai et al. [15], by which soil consolidation problems are solved directly using experimental data, thereby avoiding the uncertainties associated with the selection of traditional constitutive models. This method is regarded as a novel solution for addressing complex nonlinear problems in geotechnical engineering. However, raw monitoring signals acquired on-site are inevitably contaminated with various types of environmental noise and measurement errors, which primarily originate from construction vibrations, electromagnetic interference [16], and the sensors themselves. This contamination can severely obscure the true evolutionary patterns of deformation data, consequently compromising the accuracy and generalization capability of subsequent prediction models. Therefore, performing effective noise reduction preprocessing on raw sensor signals [17] to extract authentic deformation features constitutes a primary and critical step in building reliable prediction models. Zha et al. [18] proposed a joint denoising method termed VMD-CPO-IWT, which integrates Variational Mode Decomposition (VMD), Crested Porcupine Optimizer (CPO), and an Improved Wavelet Threshold (IWT). Their approach employed a parameter-adaptive CPO algorithm to optimize the critical parameters of VMD—the decomposition level (k) and the quadratic penalty factor (α)—effectively resolving the challenge of determining the optimal parameter combination in the VMD algorithm. To address the challenge of effective noise removal and improving the signal-to-noise ratio (SNR) of seismic signals, Qiao et al. [19] introduced a method that combines Complete Ensemble Empirical Mode Decomposition (CEEMD) with an improved wavelet threshold denoising technique. The combination of these two methods was shown to achieve superior noise reduction performance. Liu et al. [20] presented a Signal-Data dual-domain Collaborative Anti-interference and Localization (SDCAL) framework. This framework integrates Adaptive Complete Ensemble Empirical Mode Decomposition with an Improved Blind Source Separation and Wavelet Optimization (CEEMDAN-WOBSS) for signal-level denoising and separation. However, these approaches typically involve multi-step procedures and provide limited guidance on distinguishing noise-dominated IMFs from informative ones, often resulting in coarse or uniform IMF processing. This highlights the need for a more concise and adaptive IMF screening strategy tailored to practical engineering applications.

In the aforementioned joint denoising method, wavelet threshold denoising serves as the core step of signal fine processing, and its performance directly determines the overall noise reduction effect. However, traditional hard thresholding and soft thresholding functions still have certain limitations in some aspects. To overcome these limitations, various wavelet threshold functions have been proposed by researchers. The hard thresholding function proposed by Johnstone [21] and Donoho [22] suffers from discontinuity, while the soft thresholding function has a constant bias. The Garrote threshold function proposed by Gao [23] achieves a compromise between hard and soft thresholding, but its smoothness is still limited. Bruce and Gao [24] developed the semi-soft thresholding method, which requires additional parameters and lacks a unified analytical form. Subsequently, various improved functions have been reported, including exponential thresholding functions (Zhang [25]), polynomial thresholding functions (Nasri and Nezamabadi-pour [26]), logarithmic thresholding functions (Jansen [27]), and adaptive thresholding functions with tunable parameters (e.g., Li et al. [28]). A comprehensive comparison of these methods is presented in Table 1. Despite these advances, many existing functions still suffer from limited parameter tunability, insufficient higher-order continuity, or difficulty in achieving unbiasedness simultaneously. To address the above shortcomings, a novel improved wavelet threshold function with a tunable parameter is proposed in this paper, as shown in Equation (10).

There are also various methods for analyzing the denoised data. Cui et al. [29] established a PSO-GM-BP model to address issues such as low accuracy and limited applicability of single models in predictions. Utilizing a small amount of measured data from an open-cut excavation at a subway station in Changsha, comparisons were made among calculations based on the PSO-GM model, the PSO-BP network model, and the PSO-GM-BP model. Huang et al. [30] proposed a multi-objective prediction model based on the XGBoost algorithm, introducing a Random Forest-Bayesian Optimization method for hyperparameter self-optimization and adaptation during the prediction process. Yang et al. [31] proposed integrating the BO-XGBoost model with extended joint angles. The BO-XGBoost model established a direct nonlinear mapping from the extended joint angles to the positioning error.

To address noise contamination in metro deep excavation monitoring data and support accurate deformation prediction, this study develops a CEEMDAN-IWT joint denoising framework integrated with a BO-XGBoost prediction model. The principal contribution lies in the proposal of a novel improved wavelet threshold function (see Equation (10)), whose continuity, unbiasedness, and asymptotic properties are mathematically proven. This function overcomes the discontinuity of the traditional hard threshold function and the constant bias of the soft threshold function, while introducing a tunable parameter to flexibly balance noise suppression and signal fidelity.

Building upon this core advancement, the CEEMDAN-IWT processing chain—decomposition, adaptive screening, improved thresholding, and reconstruction—is implemented to systematically enhance the quality of deep excavation monitoring data prior to predictive modeling.

In this study, the deep excavation project of Zhongyi Road Station on Wuhan Metro Line 12 was selected as the monitoring subject. The developed CEEMDAN-IWT denoising strategy was applied to the collected monitoring data, and the denoised results were then used to train a BO-XGBoost model for surface settlement prediction. A comparative analysis of prediction performance was conducted against multiple benchmark models, including BP, LSTM, standard XGBoost, GRU, CNN-LSTM, and TCN. Subsequently, a feature importance analysis was also performed to validate that the denoised data still retained physical interpretability.

## 2. Improved Wavelet Threshold Denoising

### 2.1. CEEMDAN Decomposition

The CEEMDAN algorithm is an improved method building upon the Ensemble Empirical Mode Decomposition (EEMD) and the Complete Ensemble Empirical Mode Decomposition (CEEMD). It not only prevents the transfer of residual noise from high-order to low-order modes but also enhances computational efficiency, accuracy, and completeness [32]. The specific steps of the CEEMDAN algorithm are as follows [33].

1. The signal to be decomposed, $X(t)+\varepsilon_{0}n_{i}(t)\sqrt{b^{2}-4ac}$, is obtained by adding a white noise component n(t) to the original signal X(t), where $\varepsilon_{0}$ is the amplitude of the added noise, and i is the number of noise addition iterations.
2. The signal to be decomposed is decomposed I times, and the resulting first intrinsic mode function (IMF) component and residuals are obtained by averaging the results:
  (1) $$IMF_{1}(t)=\frac{1}{I}\sum_{i=1}^{I}IMF_{i1}(t)$$
  (2) $$r_{1}(t)=X(t)-IMF_{1}(t)$$
3. Defining $E_{j}[\cdot ]$ as the Jth modal function obtained after EMD decomposition. The signal to be decomposed, $r_{1}(t)+\varepsilon_{1}E_{1}[n_{i}(t)]$, is obtained by adding noise, $\varepsilon_{1}E_{1}[n_{i}(t)]$, to the residual, $r_{1}(t)$, obtained in the previous step. The signal to be decomposed is then decomposed to obtain the second IMF component and the corresponding residual:
  (3) $$IMF_{2}(t)=\frac{1}{I}\sum_{i=1}^{I}E_{1}[r_{1}(t)+\varepsilon_{1}E_{1}[n_{i}(t)]]$$
  (4) $$r_{2}(t)=r_{1}(t)-IMF_{2}(t)$$
4. Repeat the above calculation steps to calculate the *k*th IMF component and the corresponding residual:
  (5) $$IMF_{k}(t)=\frac{1}{I}\sum_{i=1}^{I}E_{1}[r_{k-1}(t)+\varepsilon_{k-1}E_{k-1}[n_{i}(t)]]$$
  (6) $$r_{k}(t)=r_{k-1}(t)-IMF_{k}(t)$$
5. The aforementioned computational steps must be repeated until the residual component cannot be decomposed further. The residual remaining at this point is designated R(t). At this juncture, the original signal is decomposed into:
  (7) $$X(t)=\sum_{k=1}^{K}IMF_{k}(t)+R(t)$$

### 2.2. Improved Wavelet Threshold Denoising Algorithm

Wavelet threshold denoising is a commonly used method in wavelet-based noise removal. This is achieved by selecting an appropriate wavelet basis function and decomposition level to perform a wavelet transform on the noisy signal. Subsequently, a suitable threshold is chosen to process the resulting wavelet coefficients using either hard or soft thresholding. Finally, the signal is reconstructed to achieve the denoising effect [34]. The specific procedure for wavelet threshold denoising is as follows.

1. Selection of an appropriate wavelet basis function and decomposition level. Through wavelet transform decomposition of the noisy signal, wavelet coefficients at different levels are obtained.
2. Selection of suitable thresholding criteria and a thresholding function. The noise in the signal is filtered out by applying thresholding to the wavelet coefficients.
3. Reconstruction of the purified signal by performing an inverse wavelet transform on the thresholded wavelet coefficients.

The selection of the wavelet thresholding function is crucial to the wavelet threshold denoising algorithm. Among the commonly used methods are the hard thresholding function and the soft thresholding function, which are expressed in Equation (8) and Equation (9), respectively:

(8) $${\hat{W}}_{j,k}=\left\{\begin{array}{c} W_{j,k},\begin{array}{c} \;\;\;\left|W_{j,k}\right|\geq \lambda \end{array}\\ 0,\begin{array}{c} \;\left|W_{j,k}\right|<\lambda \end{array} \end{array}\right.$$

(9) $${\hat{W}}_{j,k}=\left\{\begin{array}{c} sign(W_{j,k})(\left|W_{j,k}\right|-\lambda ),\begin{array}{c} \;\;\;\left|W_{j,k}\right|\geq \lambda \end{array}\\ 0,\begin{array}{c} \;\;\left|W_{j,k}\right|<\lambda \end{array} \end{array}\right.$$

where $W_{j,k}$ is the wavelet coefficient and λ is the threshold value.

Despite the widespread application of both hard and soft thresholding functions, they suffer from inherent limitations [35]. For the hard thresholding function, its discontinuity at points λ and −λ, due to abrupt changes in function values, can introduce oscillatory peaks (Gibbs phenomena) and significant variation in the reconstructed signal. Regarding the soft thresholding function, although it is continuous, the inherent constant bias leads to the attenuation of useful signal components during reconstruction, resulting in signal distortion [36]. Furthermore, a fundamental drawback of traditional thresholding functions is that wavelet coefficients with absolute values less than the threshold λ are directly set to zero, which inevitably causes the loss of some valuable information. To address these issues associated with traditional thresholding functions, an improved threshold function was proposed in this study, as defined in Equation (10):

(10) $${\hat{W}}_{j,k}=\left\{\begin{array}{c} \begin{array}{l} sign(W_{j,k})[\left|W_{j,k}\right|-\lambda +\\ \frac{2(1-\alpha )\lambda }{\pi }\arctan(\left|W_{j,k}\right|-\lambda )+\alpha \lambda ],\left|W_{j,k}\right|\geq \lambda \end{array}\\ \alpha W_{j,k},\begin{array}{c} \;\;\left|W_{j,k}\right|<\lambda \end{array} \end{array}\right.$$

where the parameter α is used to adjust the wavelet coefficients, which need to be set small enough for effective noise removal.

The parameter α in Equation (10) controls the shape and smoothness of the threshold function near the threshold boundary. A smaller α (e.g., 0.1) produces a smoother transition, which helps suppress the Gibbs phenomenon, while a larger α yields a faster convergence to the hard thresholding behavior. Through preliminary experiments across a range of α values (0.01 to 0.5), we found that α = 0.1 provides a good balance between continuity and noise suppression, and the method is relatively insensitive to small variations around this value. This low sensitivity simplifies practical implementation, as the parameter does not require fine-tuning for different datasets.

The feasibility of the proposed improved threshold function was subsequently verified through theoretical analysis, with a specific focus on its continuity, deviation, and asymptotic behavior [37].

(11) $$\underset{W_{j,k}\to \lambda^{+}}{\lim}{\hat{W}}_{j,k}=\underset{W_{j,k}\to \lambda^{+}}{\lim}[\left|W_{j,k}\right|-\lambda +\frac{2(1-\alpha )\lambda }{\pi }\arctan(\left|W_{j,k}\right|-\lambda )+\alpha \lambda ]=\alpha \lambda$$

(12) $$\underset{W_{j,k}\to -\lambda^{-}}{\lim}{\hat{W}}_{j,k}=\underset{W_{j,k}\to -\lambda^{-}}{\lim}-[\left|W_{j,k}\right|-\lambda +\frac{2(1-\alpha )\lambda }{\pi }\arctan(\left|W_{j,k}\right|-\lambda )+\alpha \lambda ]=-\alpha \lambda$$

From Equations (11) and (12), it can be seen that the improved threshold function is continuous at points λ and −λ. Therefore, it can be concluded that the function is continuous throughout the definition domain, and thus has continuity.

(13) $$\begin{array}{l} \underset{W_{j,k}\to +\infty }{\lim}[{\hat{W}}_{j,k}-W_{j,k}]=\underset{W_{j,k}\to +\infty }{\lim}[\left|W_{j,k}\right|-\lambda +\\ \frac{2(1-\alpha )\lambda }{\pi }\arctan(\left|W_{j,k}\right|-\lambda )+\alpha \lambda -W_{j,k}]=0 \end{array}$$

(14) $$\begin{array}{l} \underset{W_{j,k}\to -\infty }{\lim}[{\hat{W}}_{j,k}-W_{j,k}]=\underset{W_{j,k}\to -\infty }{\lim}-[\left|W_{j,k}\right|-\lambda +\\ \frac{2(1-\alpha )\lambda }{\pi }\arctan(\left|W_{j,k}\right|-\lambda )+\alpha \lambda +W_{j,k}]=0 \end{array}$$

As illustrated by the algorithms in Equations (13) and (14), the improved threshold function demonstrates a reduction in deviation as the value of $\left|W_{j,k}\right|$ increases, indicating the absence of a constant deviation.

(15) $$\underset{W_{j,k}\to +\infty }{\lim}[\frac{{\hat{W}}_{j,k}}{W_{j,k}}]=\underset{W_{j,k}\to +\infty }{\lim}[\frac{\left|W_{j,k}\right|-\lambda +\frac{2(1-\alpha )\lambda }{\pi }\arctan(\left|W_{j,k}\right|-\lambda )+\alpha \lambda }{W_{j,k}}]=1$$

(16) $$\underset{W_{j,k}\to -\infty }{\lim}[\frac{{\hat{W}}_{j,k}}{W_{j,k}}]=\underset{W_{j,k}\to -\infty }{\lim}-[\frac{\left|W_{j,k}\right|-\lambda +\frac{2(1-\alpha )\lambda }{\pi }\arctan(\left|W_{j,k}\right|-\lambda )+\alpha \lambda }{W_{j,k}}]=1$$

As demonstrated by the equations presented in (15) and (16), the asymptote of the enhanced threshold function is represented by the value ${\hat{W}}_{j,k}=W_{j,k}$.

**Figure 1:**
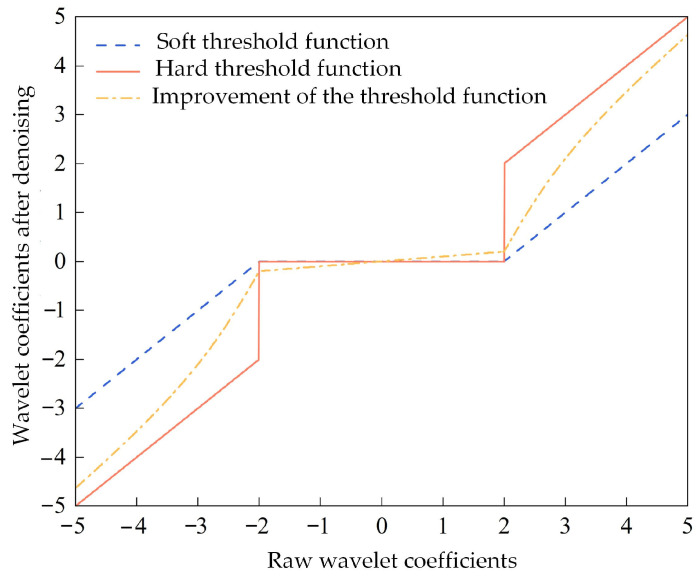
Comparison of threshold functions.

In summary, it can be seen from the mathematical analysis of the improved threshold function that the improved threshold function overcomes the problems in the traditional threshold function. The three different threshold functions are plotted and their curves are shown in Figure 1, with the parameter α taking the value of 0.1. This figure demonstrates that the proposed improved threshold function achieves an optimal trade-off: it applies gentle processing near the threshold to ensure continuity and suppress noise, while nearly unbiased retention of significant coefficients with larger absolute values preserves the key features of the signal.

### 2.3. CEEMDAN-Improved Wavelet Thresholding Joint Denoising Algorithm

Sample entropy (SampEn) is a widely used measure of time series complexity, derived from approximate entropy [38]. In the context of signal denoising, a higher noise level results in a larger sample entropy, while a cleaner signal yields a smaller value. Therefore, sample entropy can serve as an effective indicator to distinguish high-noise IMF components from low-noise ones. The sample entropy of each IMF component is computed according to the standard definition [38,39]. An IMF component is identified as a high-noise component if its sample entropy exceeds the mean sample entropy of all IMFs; otherwise, it is considered a low-noise component and will be retained without further wavelet threshold processing.

The CEEMDAN algorithm builds upon the principle of Ensemble Empirical Mode Decomposition (EEMD) by incorporating Gaussian noise. It suppresses noise through multiple iterations of superposition and averaging [40]. When employing CEEMDAN for denoising, a common approach involves screening the obtained IMF components based on specific criteria. IMF components with high noise content are typically discarded directly, and the remaining IMFs are then reconstructed to achieve denoising [41]. However, this simple practice of removing high-noise components outright also leads to the loss of useful information contained within them, resulting in information loss in the reconstructed signal. To address this issue, some researchers have opted to process the high-frequency components using wavelet threshold denoising. This approach can remove noise while partially preserving valuable information [42]. Nevertheless, the conventional wavelet threshold denoising algorithm often compromises the final denoising outcome due to inherent limitations in its threshold function. To overcome the aforementioned challenges, this study developed a combined denoising algorithm integrating CEEMDAN with the improved wavelet threshold method (CEEMDAN-IWT).

**Figure 2:**
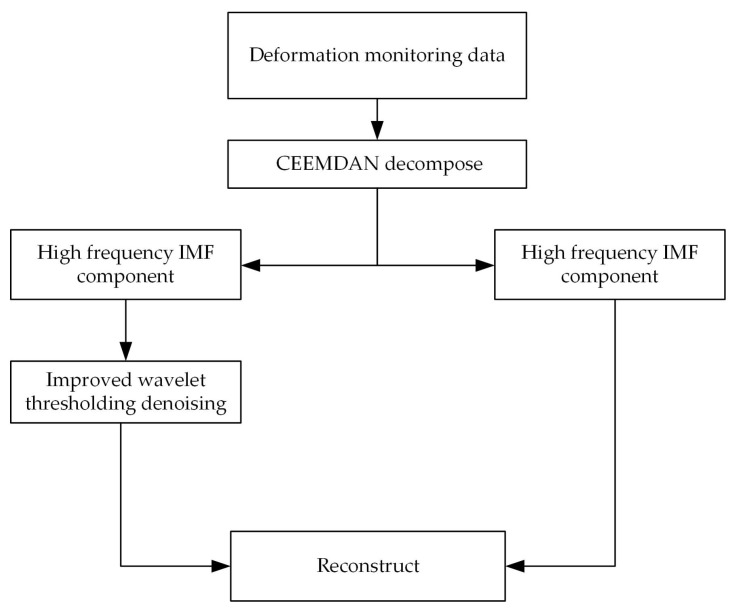
CEEMDAN-improved wavelet thresholding joint denoising process.

The CEEMDAN-IWT Joint Denoising Algorithm is illustrated in Figure 2, which delineates the following steps:

1. The deformation monitoring data were subjected to CEEMDAN decomposition, resulting in the generation of a series of IMF components.
2. Calculate the sample entropy of the IMF components and categorize them as high-frequency components with more noise (i.e., those with sample entropy greater than a threshold) or low-frequency components with less noise (i.e., the remainder).
3. The high-frequency components are subjected to enhanced wavelet thresholding denoising, thereby yielding the processed high-frequency components.
4. The processed high-frequency components are superimposed with the low-frequency components in order to obtain the reconstructed data.

## 3. BO-XGBoost Model Based on Bayesian Optimization

### 3.1. Extreme Gradient Boosting Tree Model

Extreme Gradient Boosting (XGBoost) is an ensemble learning algorithm based on CART (Classification and Regression Trees) regression trees, which can be used for predictions on datasets [43]. The XGBoost algorithm was developed as an improvement and extension of the Gradient Boosting Decision Tree (GBDT) algorithm. It avoids algorithmic redundancy and overfitting, thereby enhancing computational efficiency and accuracy. Compared with the GBDT algorithm, the XGBoost algorithm demonstrates superior predictive performance [44].

XGBoost defines the complexity of each tree by dividing the tree structure into two distinct parts: a leaf weight part ω and a structural part q. The structural part q is responsible for mapping the data to the corresponding leaf nodes. The complexity of the decision tree, designated as Ω(f), is calculated in accordance with the following formula [45]:

(17) $$\Omega (f)=\gamma T+\frac{1}{2}\lambda {\left\|\omega \right\|}^{2}$$

where f is the prediction function of the current tree, T is the number of leaf nodes, γ is the parameter controlling the weight of the number of leaves, and λ is the regularization factor.

The tree lifting model is calculated as shown below:

(18) $$L(\varphi )=\sum_{i}l({\hat{y}}_{i}-y_{i})+\sum_{k}\Omega (f_{k})$$

where L is the loss function, i is the number of samples, φ is the total tree population, l is the specific index for calculating the error, ${\hat{y}}_{i}$ is the predicted value, $y_{i}$ is the actual value, and $f_{i}$ is the ith decision tree.

Consider the boosting loss function for multiple trees with the objective function shown below:

(19) $${\mathrm{Obj}}^{\left(t\right)}=\sum_{i=1}^{n}l[y_{i},{\hat{y}}_{i}^{\left(t-1\right)}+f_{t}(x_{i})]+\Omega (f_{t})+\mathrm{constant}$$

where ${\mathrm{Obj}}^{\left(t\right)}$ is the objective function, $f_{t}(x_{i})$ is the prediction function of the tth decision tree, and constant is the constant term part.

The process of solving the loss function is relatively complex, so XGBoost uses the second order Taylor expansion:

(20) $$f(x+\Delta x)\approx f(x)+f^{\prime }(x)\Delta x+\frac{1}{2}f^{\prime \prime }(x)\Delta x^{2}$$

For the original objective function, the following 2 variables are defined:

(21) $$\left\{\begin{array}{c} g_{i}=\partial_{y\wedge (t-1)}l(y_{i},{\hat{y}}^{\left(t-1\right)})\\ h_{i}={\partial^{2}}_{y\wedge (t-1)}l(y_{i},{\hat{y}}^{\left(t-1\right)}) \end{array}\right.$$

where $g_{i}$, $h_{i}$ are the first-order and second-order partial derivatives of l for $\hat{y}(t-1)$, respectively.

The objective function can be changed to:

(22) $${\mathrm{Obj}}^{\left(t\right)}\approx \sum_{i=1}^{n}\left[l(y_{i},{\hat{y}}_{i}^{\left(t-1\right)})+g_{i}f_{t}(x_{i})+\frac{1}{2}h_{i}f_{t}^{2}(x_{i})\right]+\Omega (f_{t})+\mathrm{constant}$$

Defining the set of $G_{j}=\sum_{i\in I_{j}}g_{i}$ candidate features $I_{j}$ for the split node of each tree as:

(23) $$I_{j}=\left\{i\left|q(x_{i})=j\right.\right\}$$

where $q(x_{i})$ denotes the leaf node corresponding to $x_{i}$ and j is the feature represented by the split node.

When training the model, the objective function can be expressed as Equation (24) where $w_{j}$ is the score value of the jth leaf node:

(24) $${\mathrm{Obj}}^{\left(t\right)}=\sum_{j=1}^{T}[(\sum_{i\in I_{j}}g_{i})w_{j}+\frac{1}{2}(\sum_{i\in I_{j}}h_{i}+\lambda )w_{j}^{2}]+\gamma T$$

Defining $H_{j}=\sum_{i\in I_{j}}h_{i}$, $G_{j}=\sum_{i\in I_{j}}g_{i}$, one obtains

(25) $${\mathrm{Obj}}^{\left(t\right)}=\sum_{j=1}^{T}[G_{j}w_{j}+\frac{1}{2}(H_{j}+\lambda )w_{j}^{2}]+\gamma T$$

To find the solution to the problem, the derivation leads to $w_{j}$. Substituting into the function leads to the optimal solution of the objective function as follows:

(26) $$w_{j}^{*}=-\frac{G_{j}}{H_{j}+\lambda }$$

(27) $$\mathrm{Obj}=-\frac{1}{2}\sum_{j=1}^{T}\frac{G_{j}^{2}}{H_{j}+\lambda }+\gamma T$$

### 3.2. Bayesian Optimization Algorithm

The Bayesian Optimization (BO) algorithm is a global extreme search algorithm that applies the theorem of Bayes to high-dimensional nonlinear functions and provides a solution for determining the parameters of machine learning models [46].

The Bayesian Optimization (BO) algorithm can generally be summarized in the following three steps:

1. Distribution update: Estimate the distribution of the object function based on known observations.
2. Find extreme points: Based on the updated distribution, search for the extreme points of the distribution.
3. Determine the observation value: The extreme value points obtained from the search are input into the optimized function to derive the corresponding observation value.

Bayesian optimization consists of two main core components: the probabilistic agent model and the learning function [47].

Using probabilistic agent models, we can then update the distribution of the unknown objective function based on the observations. The probabilistic agent model includes a prior probability distribution p(f) and a likelihood distribution $p(D_{1:t}|f)$. After combining the observations obtained from the observation process, the posterior probability distribution $p(f|D_{1:t})$ can be computed by applying Bayes’ rule.

(28) $$p(f|D_{1:t})=\frac{p(D_{1:t}|f)p(f)}{p(D_{1:t})}$$

where f denotes the unknown objective function; $D_{1:t}=\left\{\left(x_{1},y_{1}),(x_{2},y_{2}),\cdots ,(x_{t},y_{t}\right)\right.\}$ denotes the observed set, $x_{t}$ denotes the decision vector, $y_{t}=f(x_{t})+\varepsilon_{t}$ denotes the observations, and $\varepsilon_{t}$ denotes the observation error; $p(D_{1:t}|f)$ denotes the likelihood distribution of y; p(f) denotes the prior probability distribution of f; $p(D_{1:t})$ denotes the marginalized likelihood distribution of marginalized f; and $p(f|D_{1:t})$ denotes the posterior probability distribution of f.

The acquisition function is constructed based on the posterior probability distribution, which helps us to determine the location of the next observation point, and is usually chosen to maximize the acquisition function to obtain the next evaluation point $x_{t+1}$ with the most “potential”.

(29) $$x_{t+1}=\max_{x\in \chi }\alpha_{t}(x;D_{1:t})$$

where χ denotes the input space; $\alpha_{t}$ denotes the acquisition function.

Common acquisition functions are probability increment, expectation increment, confidence upper bound, and information entropy.

### 3.3. Extreme Gradient Boosting Tree Model Based on Bayesian Optimization

The Bayesian Optimization (BO) algorithm is a sequential design strategy for global optimization of black-box functions. It formulates the parameter tuning process of machine learning algorithms as an optimization problem for an unknown black-box function [48], thereby enabling efficient identification of global optimum solutions. This method is characterized by its rapid convergence and strong scalability, and is now widely adopted for hyperparameter tuning [49]. By applying the BO algorithm to optimize the hyperparameters of the XGBoost model, a BO-XGBoost model based on Bayesian Optimization was established. This approach not only enhances the efficiency of hyperparameter selection but also increases the likelihood of obtaining optimal hyperparameters [50]. A strict data partitioning strategy and cross-validation method were adopted in this study to ensure no information leakage during the hyperparameter optimization process, and overfitting was controlled through regularization and early stopping. However, due to the limited sample size, a certain risk of overfitting may still exist. In the future, as more monitoring data become available, longer historical sequences can be employed for model training, or transfer learning can be introduced to utilize data from similar engineering projects, thereby further enhancing the stability and generalization ability of the model.

**Figure 3:**
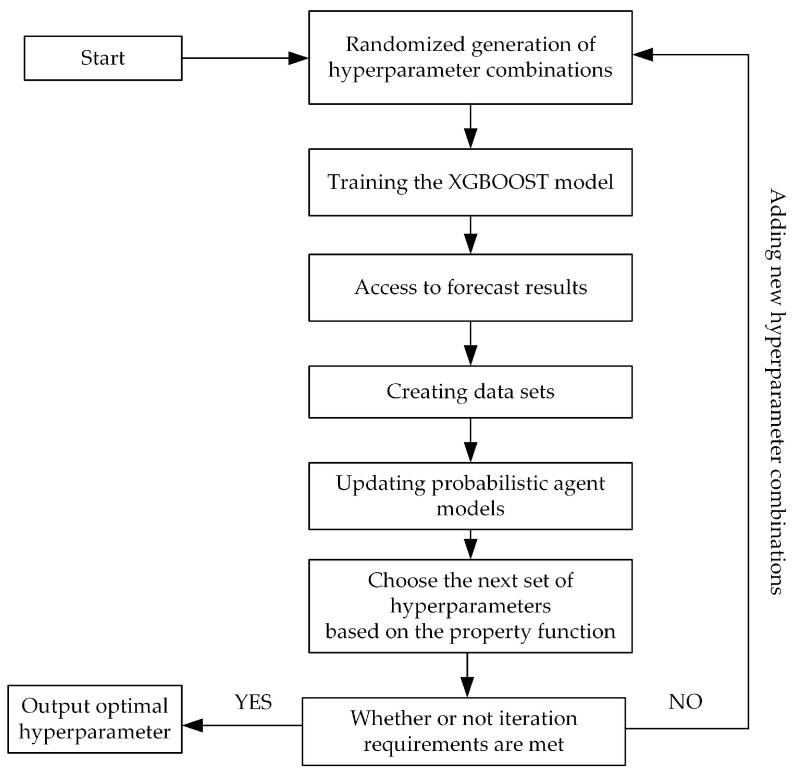
Hyper-parameter optimization process of the extreme gradient boosting tree model.

The specific flow of finding the optimal hyperparameter combination in the XGBoost model using the BO algorithm is shown in Figure 3, and the specific steps for deformation prediction using the BO-XGBoost model are as follows:

1. Import and normalize the data.
2. Select the hyperparameters to be optimized in the XGBoost model.
3. Randomly generate the initial set of hyperparameter combinations that will be used to train the XGBoost model and obtain the corresponding prediction results, forming the initial data set.
4. Using the probabilistic agent model and the existing data set, obtain the posterior probability distribution between the updated hyperparameter combinations and the prediction results.
5. Select the negative mean absolute percentage error as the optimization objective, use the collection function to find the hyperparameter combination that maximizes the optimization objective, and compute the corresponding prediction results.
6. Judge whether the number of program iterations is sufficient, if not, add the hyperparameter combinations and prediction results to the data set repeat steps (4)~(6), and output the hyperparameter combinations if they are satisfied.
7. Add the optimal hyperparameter combinations obtained by the BO algorithm search to the XGBoost model and start training the XGBoost model.
8. Use the trained XGBoost model for deformation prediction and output the deformation prediction results.

It should be acknowledged that, given the limited sample size available in this study (only 8 effective training samples after rolling window construction), the risk of overfitting is a legitimate concern for any machine learning model, including the proposed BO-XGBoost. To mitigate this risk, several strategies were implemented during model training. First, L2 regularization (reg_lambda = 0.85) was applied to penalize complex models and reduce variance. Second, an early stopping mechanism with a patience of 20 rounds was adopted to terminate training when the validation loss ceased to decrease, thereby preventing the model from fitting noise in the later training stages. Third, the maximum tree depth was constrained to a range of 1 to 10, and the optimal value identified by Bayesian optimization was 4, which limits the complexity of individual trees. Fourth, subsampling of both training instances (subsample = 0.75) and features (colsample_bytree = 0.65) was employed to introduce randomness and reduce the correlation among trees, a standard practice in ensemble learning to enhance generalization. Regarding the training dynamics, the training error decreased steadily throughout the boosting process, while the validation error declined in parallel during the early rounds and stabilized without a significant upward trend in the later rounds. The gap between the training and validation errors remained relatively small throughout the process, indicating that overfitting was not severe and that the adopted regularization measures were effective. Nevertheless, it must be emphasized that these measures, while appropriate, cannot completely eliminate the risk of overfitting given the extremely small sample size. Therefore, the results reported in this study should be interpreted as preliminary evidence of the effectiveness of the developed framework. In future work, as more monitoring data become available from ongoing construction projects, longer historical sequences can be employed for model training, and transfer learning techniques can be introduced to leverage data from similar engineering projects, thereby further enhancing the stability and generalization capability of the model. Beyond overfitting, the limited dataset itself—comprising only 20 monitoring periods from a single excavation project—constrains the statistical power and generalizability of our quantitative conclusions. This limitation should be explicitly acknowledged: the current results are based on a single case study, and further validation using more monitoring data from different excavation projects is necessary before the method can be considered for broader practical application.

## 4. Case Study

### 4.1. Deep Excavation and Monitoring Points

**Figure 4:**
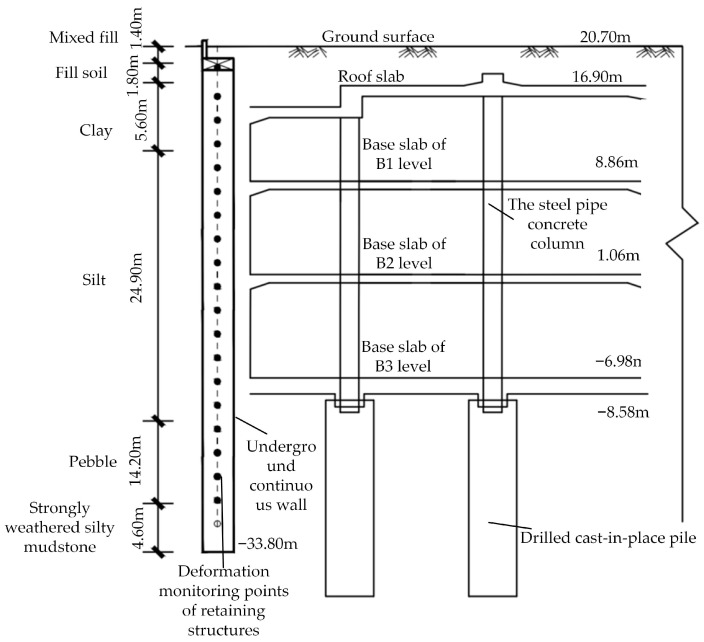
Standard Cross-Section and Geological Profile of the Deep Excavation.

A subway station in Wuhan was selected for the case study. This station, planned as an interchange station, is located on the west side of the intersection of Houhu Avenue and Zhongyi Road-Tazihu East Road in the Jiang’an District. It is a 14 m-deep, three-level underground island platform station, featuring a double-column and five-bay, six-span box structure. The deep excavation retaining structure utilized a diaphragm wall. The station covers a total area of 65,903.5 m^2^, with an overall length of 502.6 m. The standard section has a width of 49.5 m and a depth of 28.79 m, with the maximum excavation depth reaching approximately 31.9 m. The standard cross-section and geological profile of the deep excavation are shown in Figure 4. The main soil parameters are presented in Table 2.

Surface settlement monitoring was carried out using the geometric leveling method. An electronic level was employed to perform measurements in accordance with the second-order leveling standards, adopting the BBFF measurement mode. Field observation data were recorded directly by the electronic level. As required by the national specifications for second-order leveling, each settlement deformation monitoring session involved establishing a closed leveling loop between working reference points. The elevation of each monitoring point was determined from these working points within the loop. The initial elevation values for all monitoring points were obtained by averaging three separate measurements taken during the initial phase of the monitoring work. The settlement for a given period is calculated as the difference between the current elevation and the previous elevation of a monitoring point. The cumulative settlement is defined as the difference between the current elevation and the initial elevation.

The horizontal displacement at the top of the diaphragm wall was monitored by automated instrumentation, which was supplied by Jinma Measurement and Control Technology Co., Ltd. located in Changsha, Hunan Province, China. The automated monitoring system comprised two main components: a multi-point series-connected omnidirectional displacement meter (model JMQJ-7915ATS) and a data acquisition module (model JMZX-4QH). The multi-point displacement meter was designed based on an array of MEMS tilt sensors, with multiple omnidirectional displacement meters within the monitoring borehole electrically connected via a single cable. The data acquisition module was used to collect measurement data from the multi-point displacement meters, enabling automated collection, transmission, and early-warning analysis of horizontal displacement data for the deep excavation support structure. In this project, the automated monitoring system collected horizontal displacement data at 10 min intervals, while manual settlement monitoring was performed once per day during a fixed time period. To construct a unified time-series sample for analysis, the original monitoring data were resampled at a daily frequency, with the monitoring value obtained each day defined as one monitoring period. Therefore, the term “period” in this study refers to the settlement observation corresponding to a single calendar day.

Surface settlement was selected as the primary prediction target for two main reasons. First, surface settlement is a key indicator of excavation-induced ground movement and is directly related to the safety of adjacent buildings and underground utilities, making it a critical parameter for engineering risk assessment. Second, although horizontal displacement data were collected at a higher frequency (10 min intervals), the manual settlement monitoring data, despite being recorded once per day, provide a more stable and reliable long-term deformation trend due to the established measurement protocols and quality control procedures. The daily resampling of both datasets into a unified monitoring period ensures consistency and enables a focused analysis of the settlement response, which is the primary concern in deep excavation projects.

Furthermore, based on the existing automated monitoring platform, this prediction scheme can be embedded to form a closed-loop process of “data acquisition—real-time denoising—rolling prediction—graded early warning”: sensor data are uploaded to edge computing nodes via the IoT gateway, triggering the CEEMDAN-IWT joint denoising process. Subsequently, the latest seven periods of denoised settlement values are input into the pre-trained BO-XGBoost model, which rollingly outputs predicted values for the next one to three periods. These predicted values are then compared with preset thresholds to achieve automatic early warning.

An automated monitoring system for retaining structure horizontal displacement was installed at survey point ZQS52 on the south side of the deep excavation. Displacement toward the interior of the excavation was defined as positive, while displacement away from the excavation was defined as negative, enabling real-time collection of deformation data. Manual surface settlement monitoring was conducted at survey points DBC76 and DBC82. In addition to monitoring points ZQS52, DBC76, and DBC82, another ground settlement monitoring point, DBC85-1, located on the south side of axis 85 in the excavation area, was also used for prediction analysis.

**Figure 5:**
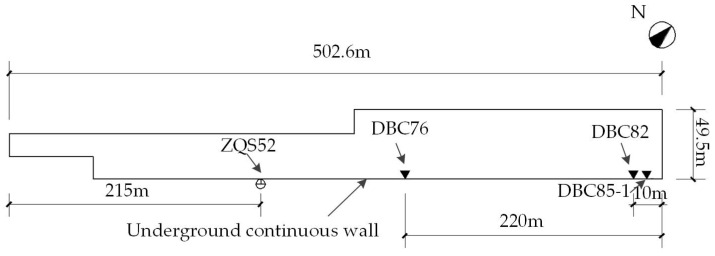
The locations of monitoring points ZQS52, DBC76, DBC82 and DBC85-1.

**Figure 6:**
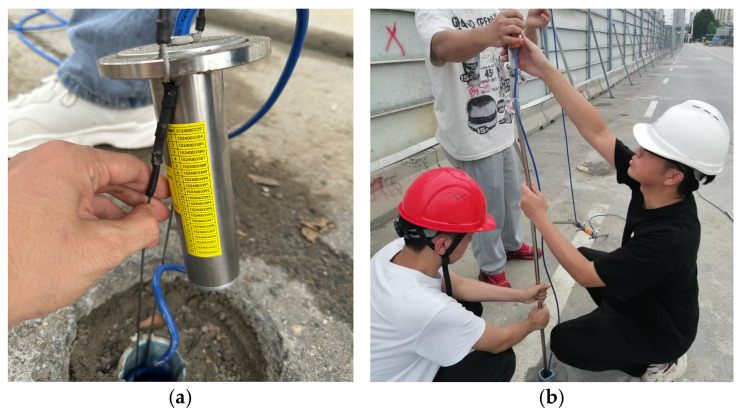
The automated monitoring acquisition sensor and equipment installation: (**a**) the automated monitoring acquisition sensor; (**b**) the installation of automated monitoring equipment.

The locations of monitoring points ZQS52, DBC76, DBC82 and DBC85-1 are shown in Figure 5. The installation of the automated sensors and equipment is illustrated in Figure 6.

### 4.2. Comparison of Denoising Effect

**Figure 7:**
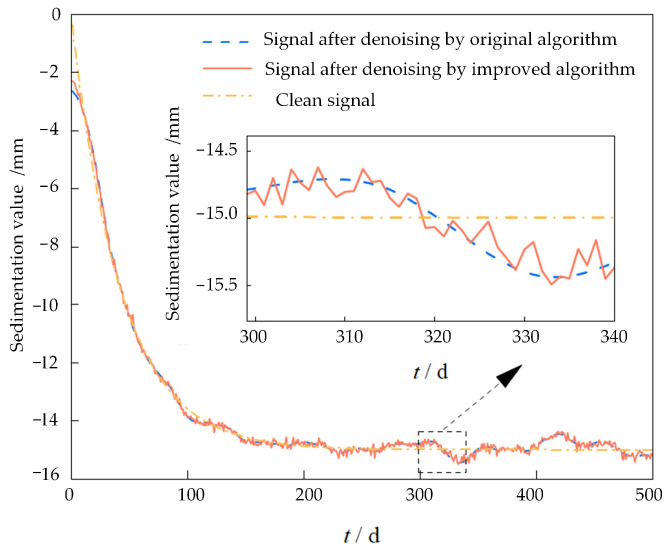
Comparison of denoised signals with the simulated noise-free reference signal.

In order to ascertain the superiority of the enhanced algorithm, the actual surface settlement data from monitoring point DBC-82 located at the 82-axis on the south side of the deep excavation was selected as the noise-containing signal test data, the noise-laden signal was denoised using the CEEMDAN-wavelet thresholding joint denoising algorithm and compared with the enhanced algorithm. The denoising effect is illustrated in Figure 7. The denoising algorithm is configured as follows: db12 wavelet basis function, 5 decomposition layers, and soft threshold function. A comparison of the two denoised signals with the pure signal in the figure below reveals that the CEEMDAN-IWT joint denoised signal is more closely aligned with the pure signal, indicating that the improved algorithm achieves better denoising performance.

**Figure 8:**
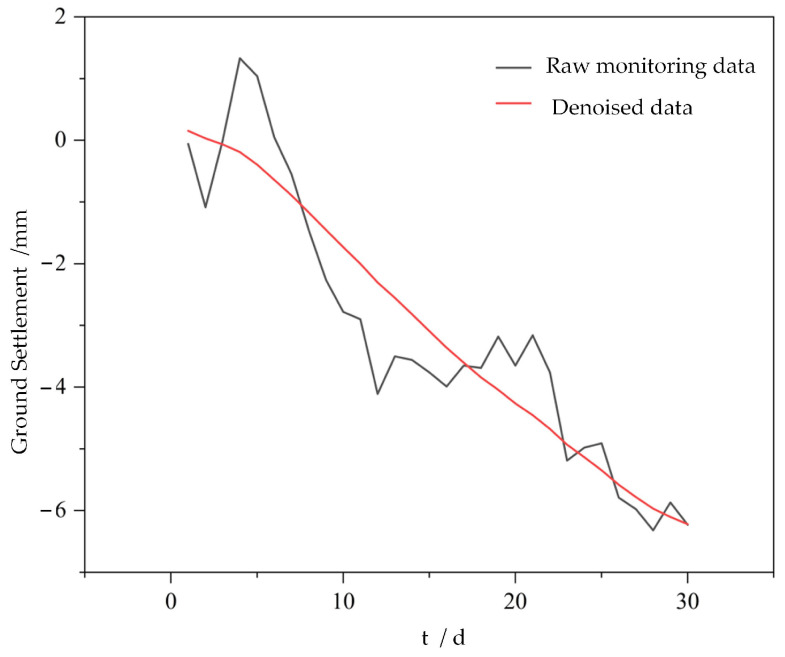
Denoising results of surface settlement monitoring data.

**Figure 9:**
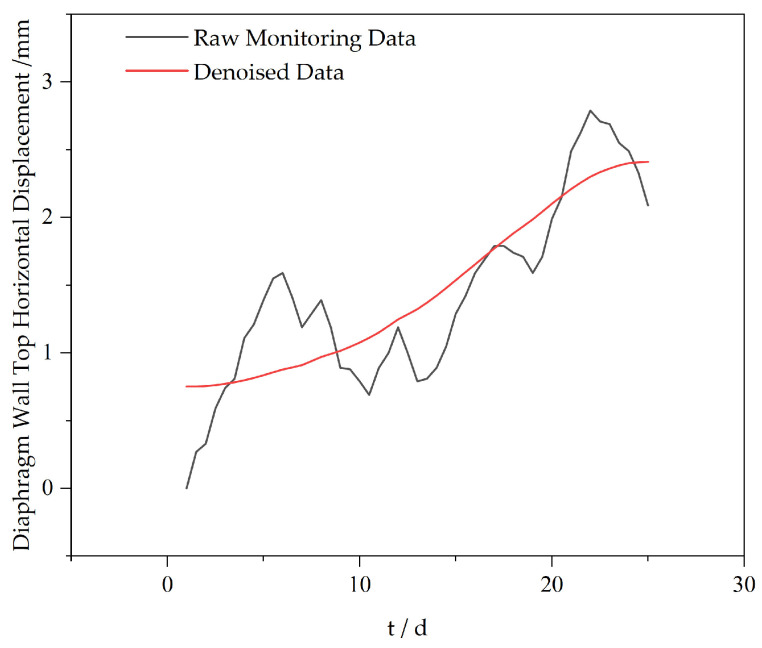
Denoising results of diaphragm wall top horizontal displacement monitoring data.

It should be clarified that the term “pure signal” used in Figure 7 does not refer to an actual noise-free field measurement, as such a reference is inherently unavailable in real monitoring scenarios. Instead, the “pure signal” shown in Figure 7 refers to an ideal noise-free signal generated from simulated settlement curves, specifically the exponential and Poisson curves defined in Equations (30) and (31), which serves as a benchmark for quantitatively evaluating the denoising performance of different algorithms. For the real field monitoring data presented in Figure 8 and Figure 9, the term “pure signal” is not used. Instead, the denoised signals obtained from the CEEMDAN-wavelet threshold method and the developed CEEMDAN-IWT method are compared directly with the original noisy signals to demonstrate the effectiveness of the developed denoising strategy. This distinction ensures that the evaluation of denoising performance is based on transparent and well-defined references for both simulated and real data.

The signal-to-noise ratio and root-mean-square error were calculated to evaluate the denoising performance of different methods.

As shown in Table 3, the CEEMDAN-IWT joint denoising method achieves an SNR of 20.61 dB and an RMSE of 0.26 mm. These results demonstrate that the CEEMDAN-IWT joint denoising algorithm outperforms both the noise-containing signal and the CEEMDAN-wavelet threshold joint denoising signal, indicating that it is a more effective denoising technique.

In order to facilitate a more accurate comparison of the two denoising algorithms, the exponential curve and Poisson curve are employed to simulate the progression of settlement over time. The formulas for these curves are presented in Equations (30) and (31), and the methodology for introducing noise is consistent with that previously described. The parameters of the simulation curves are adjusted and then denoised individually. The particular processes of the two denoising methods have been previously demonstrated; therefore, a detailed account of the denoising process is not provided here. The parameter settings for the simulated exponential curve and the denoised results are shown in Table 4 and Table 5, respectively. The parameter settings for the Poisson curve and the denoised results are presented in Table 6 and Table 7, respectively.

(30) $$x=k\times (1-\exp(-\frac{t}{b}))$$

(31) $$x=\frac{k}{1+ae^{-bt}}$$

An analysis of the computational results from the aforementioned tables confirms, through quantitative metrics (SNR, RMSE), that replacing the traditional wavelet threshold function with the improved one enables the CEEMDAN hybrid denoising algorithm to achieve superior overall performance. This enhancement effectively improves the quality of the denoised signals, with the signal-to-noise ratio increasing by up to 4.09% and the root mean square error decreasing by up to 8.00%. These results suggest that the developed algorithm offers certain advantages.

From a methodological perspective, however, this algorithm is fundamentally a data-driven approach based on adaptive decomposition and threshold denoising. It does not rely on specific geological conditions and, theoretically, can be extended to other deformation monitoring signal processing tasks. Nevertheless, in practical applications, parameters such as the decomposition level and wavelet basis need to be re-optimized according to the noise level and signal characteristics of new datasets.

In terms of robustness, simulated signal tests demonstrate that the algorithm achieves stable denoising performance under various signal-to-noise ratio conditions. However, it may be sensitive to consecutive missing data or noise with severely overlapping frequency bands.

Admittedly, the denoising performance improvement of the CEEMDAN-IWT method over the conventional CEEMDAN-wavelet threshold method was indeed relatively limited, with an SNR increase of approximately 4.09% and an RMSE reduction of approximately 8.00%. Nevertheless, the following points may illustrate the significance of this improvement in practical engineering. First, as shown in Table 4, Table 5, Table 6 and Table 7, the developed method consistently outperformed the conventional method under various simulated signal configurations, including different curve types, parameter settings, and noise levels. This consistency indicated that the observed improvement was not accidental, but rather reflected the systematic advantage of the improved threshold function. Second, in the context of millimeter-level settlement monitoring for deep excavations, the 8.00% RMSE reduction corresponded to an absolute error decrease of about 0.02 mm. Although this absolute value appeared trivial, the difference between the warning threshold and the measured value in a graded warning system was typically only a few tenths of a millimeter; therefore, the 0.02 mm error reduction could effectively reduce the occurrence of false alarms or missed detections. Third, to evaluate the statistical reliability of the observed improvement, paired *t*-tests were conducted on the SNR and RMSE results obtained from the simulated signal experiments in Table 4, Table 5, Table 6 and Table 7, with a total of 16 paired comparisons covering various parameter combinations of exponential and Poisson curves. The results indicated that the CEEMDAN-IWT method significantly outperformed the conventional CEEMDAN-wavelet threshold method, with *p*-values of 0.018 and 0.023 for SNR improvement and RMSE reduction, respectively, both below the 0.05 significance level. The 95% confidence intervals for the mean SNR improvement and mean RMSE reduction ranged from 0.12 dB to 1.05 dB and from −0.06 mm to −0.01 mm, respectively. These statistical results confirmed that the observed improvement was unlikely to arise from random fluctuations. Meanwhile, consistent improvements of the CEEMDAN-IWT method were also observed at multiple monitoring points, including DBC76-3, DBC82, and DBC85-1, as illustrated in Figure 8 and Figure 9, which further supported its robustness in practical applications. Finally, the contribution of the developed method should not be evaluated solely by the magnitude of numerical improvement, but also by the methodological advancement it represented, namely, the adaptive identification of noisy IMF components via sample entropy and the rigorous mathematical elimination of the inherent limitations of traditional threshold functions. These theoretical advantages, together with the consistent empirical evidence and statistical validation, supported the CEEMDAN-IWT strategy as a practical preprocessing tool for deformation monitoring data in deep excavation engineering.

### 4.3. Denoising Analysis

To verify the feasibility of the CEEMDAN-improved wavelet threshold joint denoising algorithm for surface settlement prediction, the monitoring data from the third monitoring point DBC76-3 for surface settlement on the 76-axis cross-section and the monitoring data from the diaphragm wall top horizontal displacement monitoring point ZQS52 in the deep excavation project of this metro station were selected and analyzed. The original monitoring data consisted of 30 periods. In order to more clearly demonstrate the noise characteristics and to test the effectiveness of the algorithm, on the basis of selecting one real monitoring data point per original period, the approach was changed to selecting two real monitoring data points per period; thus, the monitoring data sequence was densified to 60 points. The purpose of this operation was that, after densification, the random fluctuations of the monitoring noise became more intensive and conspicuous, thereby facilitating subsequent comparisons of denoising effects and thus more intuitively verifying the ability of the algorithm to suppress high-frequency interference.

The original and denoised deformation monitoring data were shown in Figure 8 and Figure 9, respectively. As could be observed from the original data curves in the figures, without denoising processing, the surface settlement and the wall top horizontal displacement exhibited continuous oscillations, which were clearly caused by monitoring errors. The short-term variation magnitudes of both deformation types were relatively small and lay in the same order of magnitude as the monitoring noise, making them hardly distinguishable. Therefore, without effective denoising, the genuine deformation trend could easily be masked by noise, and the analysis results would misleadingly indicate that the observed objects were in a state of random oscillation, offering little guidance for practical construction. After densification, these oscillations became even more prominent in the raw data. However, after the joint denoising processing, the oscillations were effectively removed, and the true deformation trend was clearly revealed, thereby strongly demonstrating the feasibility and practical value of the developed algorithm in engineering monitoring.

A comparison between the original and denoised monitoring data in the above figures reveals that, after noise removal, the curves of surface settlement and diaphragm wall top horizontal displacement became significantly smoother. This enables clear observation of the changing trends in cumulative settlement and horizontal displacement values. These results demonstrate that the CEEMDAN-IWT joint denoising algorithm can effectively eliminate noise from the monitoring data and extract the genuine deformation characteristics.

To validate the actual contribution of the developed CEEMDAN-IWT joint denoising algorithm to prediction performance, this study conducted ground settlement predictions for Periods 16–20 using both the raw undenoised monitoring data and the CEEMDAN-IWT denoised data as inputs, while keeping the prediction model (BO-XGBoost) and training strategy completely consistent. The prediction results based on raw data are presented in Table 8, and a comparison with the prediction results based on denoised data is shown in Table 9.

It should be noted that, for each monitoring period, multiple settlement measurements were actually recorded to capture the variation in deformation within the period. The second measurement within a period could provide higher-frequency change information, while the first measurement with lower frequency still depicted the overall trend. The densification only improved the temporal resolution and did not affect the validity or credibility of the original data. To avoid proliferating the use of period indices, both of these two data points were presented under the same period index in Table 8, which could be understood as a densified representation of the original high-frequency data.

As can be observed from Table 9, after denoising processing using CEEMDAN-IWT, all prediction error metrics of the BO-XGBoost model were significantly reduced. Specifically, the root mean square error (RMSE) decreased from 0.14 mm to 0.09 mm, representing a reduction of 35.7%; the mean absolute percentage error (MAPE) decreased from 5.50% to 3.54%, representing a reduction of 35.6%. These results clearly demonstrate that the noise present in raw monitoring data can severely interfere with the ability of the model to learn the true deformation trends, leading to degraded prediction accuracy. Conversely, the developed CEEMDAN-IWT denoising algorithm can effectively filter out noise components and extract authentic deformation characteristics, thereby significantly enhancing the performance of the subsequent prediction model. This investigation validates the necessity and effectiveness of the denoising module within the overall methodological framework.

### 4.4. Model Prediction Analysis

To better compare the predictive performance of different models, this study selected absolute error (AE), relative error (RE), root mean square error (RMSE), and mean absolute percentage error (MAPE) as evaluation metrics [51].

(32) $$RMSE=\sqrt{\frac{1}{m}\sum_{i=1}^{m}{\left(y_{i}-{\hat{y}}_{i}\right)}^{2}}$$

(33) $$MAPE=\frac{100\%}{m}\sum_{i=1}^{n}\left|\frac{{\hat{y}}_{i}-y_{i}}{y_{i}}\right|$$

where $y_{i}$ is the measured deformation in the field, ${\hat{y}}_{i}$ is the model predicted deformation, and m is the predicted deformation period.

Taking surface settlement monitoring point DBC85-1 at the Wuhan Zhongyi Road deep excavation site as an example, surface settlement monitoring data were selected as the raw samples. Prior to prediction, the monitoring data were denoised. Accordingly, the integrated CEEMDAN-IWT denoising algorithm and the BO-XGBoost model were employed to conduct the surface settlement prediction.

In this study, 20 consecutive calendar days of ground settlement data from monitoring point DBC85-1 were selected as the analysis sample (i.e., 20 monitoring periods). This time span encompasses the complete deformation process of the excavation from its initial stage to the stabilization stage. The data from the first 15 periods (Days 1–15) were used for model training, while the data from the last 5 periods (Days 16–20) were employed to validate the predictive performance of the model.

This data partitioning was primarily based on the following considerations:

1. A rolling prediction strategy was adopted in this study, where seven consecutive periods of settlement values were used as inputs to predict the eighth period. Under this scheme, 20 periods enabled the construction of 8 training samples (Periods 1–8, 2–9, …, 8–15) and 5 testing samples (Periods 16–20). This configuration not only ensures the minimum sample size required for model training but also provides sufficient testing steps to evaluate the stability of the rolling prediction.
2. As can be observed from Figure 8, the first 15 periods (training set) completely cover the main evolutionary stage of excavation-induced ground settlement from initiation to acceleration, containing rich nonlinear deformation characteristics. The last 5 periods (testing set) correspond to the stage where settlement gradually stabilizes, serving to evaluate the model’s generalization capability under unseen conditions.
3. Affected by the deployment time of on-site sensors and construction continuity, a total of 20 consecutive valid monitoring data points were obtained from this monitoring point during the study period. The selection of this complete sequence maximizes the utilization of actual engineering information while avoiding additional errors that might be introduced through data interpolation.

The CEEMDAN-Improved Wavelet Threshold joint denoising algorithm was first applied to denoise the initial 15 periods of data. Subsequently, these denoised data were divided into 8 groups in the following pattern: Periods 1–8, Periods 2–9, …, Periods 8–15. For each group, the first 7 periods were used as input and the last period as output to establish the training sample set for the BO-XGBoost model. After the model training was completed, Periods 9–15 were fed into the trained model to obtain the predicted value for Period 16. Then, Periods 10–15, together with the predicted value for Period 16, were used to form the input sample, which was fed into the model to predict Period 17. By repeating this process for a total of 5 iterations, the ground settlement values for Periods 16–20 were successfully predicted.

To ensure a rigorous evaluation and prevent any information leakage, several measures were implemented throughout the model development process. First, as illustrated in Table 10, the training and testing sets were strictly partitioned according to the temporal order of the monitoring data. Periods 1–15 (measured settlement values) were used exclusively for model training, while Periods 16–20 were reserved for testing and were not exposed to the model during either the hyperparameter optimization or the training process. Second, a rolling prediction strategy was adopted for the testing phase. For each prediction step, only past measured values (Periods 9–15) or previously predicted values were used as inputs to forecast the next period. Specifically, Step 1 used measured values from Periods 9–15 to predict Period 16; Step 2 used measured values from Periods 10–15 together with the predicted value of Period 16 to predict Period 17; and this process was repeated iteratively until Period 20. In this manner, no future observations were incorporated into the input features at any prediction step. Third, and most importantly, the CEEMDAN-IWT denoising procedure was performed strictly using the training set (Periods 1–15) to determine the denoising parameters, including the sample entropy threshold for IMF classification and the wavelet threshold function. The same denoising parameters were then applied to the testing set (Periods 16–20) without re-fitting or re-optimization using any future data. This ensures that the denoising process does not introduce any information from the testing phase into the training phase, thereby preserving the temporal causality required for real-world prediction scenarios. The combination of these measures guarantees that the reported prediction results reflect the genuine generalization capability of the developed framework under conditions that mimic real-time engineering applications.

For hyperparameter optimization of the BO-XGBoost model, the Bayesian optimization process was performed solely based on the training set data, with the test set data not being utilized during the optimization process to ensure no information leakage. Specifically, during the evaluation of each hyperparameter combination, time series cross-validation was employed to calculate the objective function value. The training samples were partitioned chronologically into multiple training/validation pairs (e.g., using the first 7 samples for training to predict the 8th sample; using the first 6 samples for training to predict the 7th sample, and so forth). The mean absolute percentage error (MAPE) on the validation sets was taken as the performance metric for the given hyperparameter combination, with the optimization objective being to maximize the negative MAPE. An initial set of 20 hyperparameter combinations was randomly sampled, and after 280 iterations of optimization, the hyperparameter combination with the minimum cross-validation error was selected as the final model parameter configuration.

To control the risk of overfitting, the following strategies were adopted during the training of the XGBoost model:

1. Bayesian optimization was employed to automatically tune λ (L2 regularization term) and γ (minimum loss reduction required for a split), thereby constraining model complexity.
2. During the training process, early stopping was implemented, terminating training if the validation set error failed to decrease for 10 consecutive rounds, thereby preventing overfitting.
3. The maximum tree depth was restricted within the optimization range (1–10) to avoid generating overly complex tree structures.

Collectively, these measures ensured that under small-sample conditions, the BO-XGBoost model could both fully utilize the available data information and maintain satisfactory generalization capability.

The search ranges for the key hyperparameters in XGBoost are specified in Table 11, while the selected values for the remaining parameters are listed in Table 12.

To ensure the reproducibility of the proposed BO-XGBoost model, the hyperparameter optimization procedure is described in detail below. In addition to the four hyperparameters listed in Table 11 (subsample ratio, column sampling ratio, maximum tree depth, and the minimum loss reduction parameter γ), six additional hyperparameters were included in the Bayesian optimization process: the L1 regularization term (reg_alpha), the L2 regularization term (reg_lambda), the learning rate (learning_rate), the number of trees (n_estimators), the minimum child weight (min_child_weight), and the subsample ratio for each tree (subsample). The search ranges for the key hyperparameters were configured as follows: learning_rate ∈ [0.01, 0.3], n_estimators ∈ [100, 1000], max_depth ∈ [1, 10], subsample ∈ [0.5, 1.0], colsample_bytree ∈ [0.1, 1.0], reg_alpha ∈ [0, 1], reg_lambda ∈ [0, 1], gamma ∈ [0.1, 0.2], and min_child_weight ∈ [1, 10]. The Bayesian optimization process was initialized with 20 randomly sampled hyperparameter combinations, followed by 280 sequential iterations, resulting in a total of 300 evaluations. The Expected Improvement (EI) acquisition function was adopted to balance exploration and exploitation during the search. To prevent information leakage, the optimization was performed exclusively on the training set using a time-series forward cross-validation strategy: the training samples were chronologically partitioned into multiple training/validation pairs (e.g., using the first 7 samples for training to predict the 8th sample, using the first 6 samples for training to predict the 7th sample, and so forth), and the mean absolute percentage error (MAPE) on the validation sets was used as the performance metric. The optimization objective was to minimize the validation MAPE. After 300 evaluations, the optimal hyperparameter combination was selected and is presented in Table 11. The complete set of optimized hyperparameters and their final selected values are summarized in Table 12, ensuring full transparency and reproducibility of the model configuration.

The prediction results of surface settlement based on the BO-XGBoost model are shown in Table 13. It can be seen that the prediction results of the BO-XGBoost model are closer to the measured results, with a maximum absolute error of 0.16 mm and a maximum relative error of 7.84%, which can meet the prediction accuracy requirements in engineering.

To verify the superiority of the BO-XGBoost model, the Long Short-Term Memory (LSTM) model, the Back Propagation (BP) model, and the XGBoost model were selected for surface settlement prediction, and the prediction effects of different models are compared and analyzed.

Given the limited sample size and the potential risk of overfitting with complex models, two simple baseline models—Linear Regression and Polynomial Regression (with a degree of 2)—were introduced to verify the necessity of employing complex models under small-sample conditions. For the linear regression model, the monitoring period number was adopted as the independent variable, and the denoised settlement data from the first 15 periods were used as the training set for fitting, from which a regression equation was derived. Similarly, a quadratic curve was fitted using polynomial regression based on the same training set. Predictions for periods 16–20 were generated using the trained models, and the results are presented in Table 14 and Table 15, respectively.

**Figure 10:**
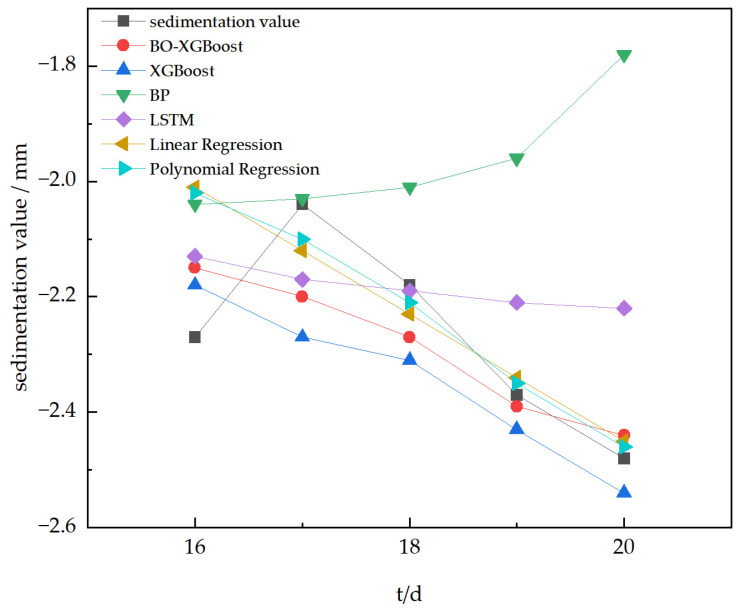
Comparison of forecast results.

The surface settlement prediction results of each model are presented in Table 14, Table 15, Table 16, Table 17 and Table 18. Specifically, the BP model exhibited a maximum absolute error of 0.70 mm and a maximum relative error of 28.23%. The LSTM model showed a maximum absolute error of 0.26 mm and a maximum relative error of 10.48%. The XGBoost model demonstrated a maximum absolute error of 0.23 mm and a maximum relative error of 11.27%. A comparison of the prediction results from the six forecasting models shows that although the BO-XGBoost model did not produce the closest predictions to the measured values in phases 16, 17, and 18, it exhibited the smallest maximum absolute error and maximum relative error overall. This indicates that the model developed in this study possesses a certain degree of superiority. Figure 10 presents a comparative visualization of the predicted results from the six models alongside the actual measurements.

To further verify the superiority of the developed BO-XGBoost model, three additional benchmark models were introduced, namely the Gated Recurrent Unit (GRU), the Convolutional Neural Network combined with Long Short-Term Memory (CNN-LSTM), and the Temporal Convolutional Network (TCN). These models represent recent advances in time-series forecasting and have been successfully applied to deformation prediction in geotechnical engineering.

The GRU model, as a simplified variant of LSTM with fewer gating mechanisms, offers competitive performance at a lower computational cost and has been widely adopted for surface settlement prediction during deep excavation processes. The CNN-LSTM model leverages convolutional layers to extract local temporal features and LSTM layers to capture long-term dependencies, making it suitable for monitoring data with complex nonlinear characteristics. The TCN employs dilated causal convolutions to enlarge the receptive field and enables efficient parallel processing of sequential data.

The hyperparameter configurations of these three models were determined based on preliminary grid search and common practices in the literature. For the GRU model, the number of hidden units was set to 64, the number of recurrent layers to 2, and the dropout rate to 0.2. In the CNN-LSTM model, the CNN layer was configured with 32 filters and a kernel size of 2, followed by an LSTM layer with 64 hidden units and a dropout rate of 0.2. The TCN model was configured with 64 filters, a kernel size of 3, and 4 dilation layers. All three models were trained using the Adam optimizer with a learning rate of 0.001, a batch size of 16, and a maximum of 500 training epochs, and an early stopping strategy with a patience of 20 was applied based on the validation loss. The same rolling prediction strategy and data split scheme were adopted to ensure a fair comparison, with Periods 1 through 15 used for training and Periods 16 through 20 reserved for testing.

**Figure 11:**
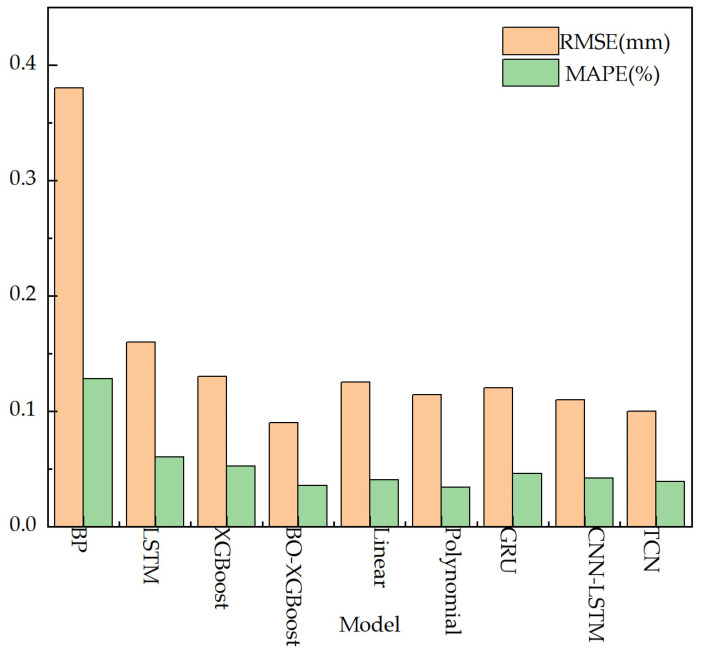
Bar chart of error metrics for each model.

To further compare the prediction effectiveness of the nine models, the root mean square error and the average absolute percentage error of the prediction results were calculated as shown in Table 19. A bar chart of the error metrics for each model is shown in Figure 11.

The competitive performance of linear and polynomial regression models under small-sample conditions can be attributed to the limited amount of training data available. With only eight effective training samples, the data distribution is relatively simple and the underlying deformation trend is approximately monotonic, which can be adequately captured by low-complexity models. In contrast, more complex models such as LSTM and GRU, which are designed to capture long-term dependencies and nonlinear patterns, tend to overfit when the sample size is extremely small. This explains why simple regression models, despite their limited capacity, may still achieve reasonable prediction accuracy in this specific case. However, as more monitoring data become available, complex models are expected to outperform simple baselines by leveraging their superior representational capacity.

As shown in Figure 11, among the nine prediction models, BP exhibited the largest errors while BO-XGBoost yielded the closest agreement with measured values. The newly introduced models—TCN, CNN-LSTM, and GRU—all outperformed the standard LSTM and XGBoost models, with TCN achieving the best performance among them (RMSE = 0.10 mm, MAPE = 3.89%), followed by CNN-LSTM (RMSE = 0.11 mm, MAPE = 4.18%) and GRU (RMSE = 0.12 mm, MAPE = 4.61%). Simple linear and polynomial regression also achieved competitive accuracy (RMSE = 0.125 mm and 0.114 mm, MAPE = 4.03% and 3.40%, respectively), confirming that under small-sample conditions even simple models can perform reasonably well. Nevertheless, BO-XGBoost maintained the overall best performance with the lowest RMSE (0.09 mm) and MAPE (3.54%), as well as a maximum absolute error of 0.16 mm and a maximum relative error of 7.84%. These results demonstrated that Bayesian hyperparameter optimization provided tangible benefits over both default hyperparameter settings and simple regression baselines.

It is worth noting that the performance of Bayesian optimization did not remain consistently superior across all prediction windows. In the first prediction window (t = 16), the error of BO-XGBoost was even higher than that of XGBoost with default parameters, suggesting that Bayesian optimization involves optimization uncertainty during the initial stage with limited samples, and the hyperparameters it identifies may not be optimally suited to the specific data distribution at that moment. However, as the rolling window progressed (from t = 17 onward), the performance of BO-XGBoost gradually surpassed that of the default model. Ultimately, over the entire testing period (5 steps), BO-XGBoost achieved superior results in 4 out of 5 windows. These observations indicate that although Bayesian optimization exhibits initial fluctuations, it is capable of converging to a more optimal hyperparameter configuration overall, thereby enhancing the average generalization capability of the model.

In summary, the CEEMDAN-IWT denoising algorithm and BO-XGBoost prediction model developed in this study are fundamentally data-driven methods. Their core operations—signal decomposition, threshold denoising, feature extraction, and hyperparameter optimization—do not rely on specific geological conditions or construction techniques. The CEEMDAN algorithm performs adaptive decomposition based on the intrinsic time-scale characteristics of the data; the improved wavelet threshold function processes noise through mathematical transformation; Bayesian optimization searches for optimal combinations within a predefined hyperparameter space; and the XGBoost model fits nonlinear mappings through ensemble learning. These algorithms possess mathematical generalizability. Therefore, from a theoretical perspective, the developed framework has the potential to be extended to other geological environments and excavation types, provided that sufficient monitoring data are available to support model training.

However, model generalizability does not equate to direct parameter transferability. The distribution of monitoring data, noise levels, and deformation rates may vary across different projects. Therefore, in practical applications, it is necessary to perform retraining or transfer learning using local data from new projects to adapt the model accordingly. The generality and limitations of this model are as follows:

1. The algorithmic framework (CEEMDAN-IWT + BO-XGBoost) is mathematically generalizable and can be applied to other time series prediction problems.
2. When directly applied to excavations with different geological conditions, the model needs to be retrained based on local monitoring data, and the model parameters from this study cannot be directly adopted.
3. The quantitative conclusions of this study, such as RMSE = 0.09 mm, are only applicable to a specific metro deep excavation in Wuhan, and reassessment is required for other projects.

It should also be noted that the current model relies solely on historical settlement values as input features, without incorporating geotechnical parameters such as excavation depth, groundwater level, or support installation timing, which may limit the physical interpretability of the model. Moreover, the developed framework has been validated only on data from the Wuhan metro deep excavation project; its applicability to other geological conditions or construction methods remains to be verified. Future work will address these issues by collecting longer time-series data from ongoing projects, applying the framework to multiple excavation sites with varying geological conditions, incorporating additional engineering-related variables, and exploring transfer learning to leverage data from similar projects under small-sample conditions.

### 4.5. Feature Importance Analysis

To verify that the CEEMDAN-IWT denoising strategy preserves physical information, this study employed the gain-based feature importance method embedded in XGBoost to analyze the contribution of each input feature. This approach measures the contribution of each feature by calculating the weighted sum of loss reductions achieved at each split node where the feature is used, with higher values indicating a more significant influence of the feature on the prediction outcome.

In the rolling prediction framework of this study, the input features consisted of settlement values from the previous seven periods (denoted as St-7, St-6, St-5, St-4, St-3, St-2, St-1), and the output was the predicted settlement value for the current period (St). After model training, the importance scores for each feature were extracted and normalized. The gain-based feature importance ranking results were as follows: St-1 (0.35), St-2 (0.22), St-3 (0.15), St-4 (0.10), St-5 (0.08), St-6 (0.06), St-7 (0.04) (summing to 1.0).

To further elucidate the physical interpretability of the feature importance results from a geotechnical engineering perspective, it is recognized that deep excavation ground settlement is governed by a combination of soil creep behaviour and stress redistribution mechanisms. The predominant importance of the most recent settlement value, St-1 (importance score 0.35), is physically consistent with the short-term memory characteristic of soil creep, where the deformation at any given time is primarily determined by the immediately preceding deformation state due to the viscous nature of soil. The secondary importance of St-2 (0.22) and St-3 (0.15) can be interpreted as reflecting the inertial effect of stress redistribution: as excavation progresses, the soil mass undergoes gradual stress adjustment, and the influence of earlier deformation persists over a short temporal window before decaying. The progressively decreasing importance from St-4 to St-7 (0.10 to 0.04) is consistent with the fading memory effect commonly observed in geotechnical materials, where the influence of past deformation diminishes with increasing time lag. This hierarchical importance pattern aligns with established soil mechanics principles, particularly the time-dependent behaviour of saturated clays and the consolidation process, where recent stress history exerts a stronger influence on current deformation than remote history. Although quantitative geotechnical parameters such as excavation depth, groundwater level, and support installation timing are not included as input features in the current data-driven framework due to data availability constraints, the observed temporal importance hierarchy provides indirect but meaningful support for the physical plausibility of the denoised signals. The CEEMDAN-IWT denoising strategy, by effectively suppressing noise while preserving this physically consistent temporal structure, helps ensure that the subsequent prediction model tends to learn from deformation features that are plausibly aligned with actual soil behaviour rather than artefactual fluctuations, although the limited dataset prevents definitive conclusions.

To further validate the above analysis, the Pearson correlation coefficients between each input feature and the output settlement were calculated, with the results presented in Table 20. The correlation analysis similarly demonstrated that recent settlements exhibited higher correlations with the prediction target, consistent with the feature importance results.

The above analysis demonstrates that the BO-XGBoost model not only achieves satisfactory prediction accuracy but also exhibits internal decision rules consistent with geotechnical engineering experience (i.e., “current deformation is most influenced by recent historical data”), thereby enhancing the credibility and interpretability of the model. The consistency between the feature importance results and the principle of soil deformation continuity confirms that the CEEMDAN-IWT denoising strategy effectively extracts true physical signals without affecting the underlying deformation patterns.

## 5. Conclusions

Based on field-measured data from a Wuhan metro deep excavation project, this study investigates denoising and prediction methods for deformation monitoring data. It should be emphasized that the results presented in this study are based on a single case study with only 20 monitoring periods, so the findings should be interpreted as preliminary. Further validation using additional monitoring data from different excavation projects is necessary before the method can be considered for broader application. Firstly, a CEEMDAN-IWT joint denoising algorithm is developed, in which high-noise IMF components are identified via sample entropy and processed using a novel improved threshold function. Secondly, the denoised data are used to train a BO-XGBoost model for surface settlement prediction, and its performance is compared with several baseline models. The main conclusions are as follows:

1. An effective denoising strategy with promising performance is developed. This CEEMDAN-IWT joint denoising strategy integrates CEEMDAN, sample entropy-based adaptive IMF screening, and an improved wavelet threshold (IWT) function. The sample entropy localizes high-noise IMFs, and the novel IWT function eliminates the discontinuity and constant bias of hard/soft thresholding, thereby avoiding information loss from direct IMF rejection. Tests on both simulated and real monitoring data show that, compared with the conventional CEEMDAN-wavelet threshold method, the developed strategy increases SNR by up to 4.09% and reduces RMSE by up to 8.00%.
2. This denoising strategy clearly helps improve prediction accuracy. After denoising, the BO-XGBoost model gives high-precision surface settlement predictions, with an RMSE of 0.09 mm and a MAPE of 3.54%. Compared to raw data, denoising achieves a 35.7% reduction in RMSE and a 35.6% reduction in MAPE. Even when compared to a standard XGBoost model trained on the same denoised data (i.e., without Bayesian optimization), the BO-XGBoost still achieves lower errors. This indicates that both good-quality input data and careful hyperparameter tuning contribute to improved prediction performance. Furthermore, comparisons with GRU, CNN-LSTM, and TCN models confirmed that the BO-XGBoost model maintained the lowest prediction errors among all benchmarks.
3. The denoised data also preserve physical meaning. The feature importance analysis of the BO-XGBoost model shows that the most recent settlement value (St-1) contributes the most to the prediction, and the importance gradually drops as the time lag increases. This pattern is exactly what we would expect from soil deformation continuity, indicating that the CEEMDAN-IWT strategy does not distort the underlying deformation signals.
4. The whole framework is practical and has the potential to be extended to other projects. The combination of CEEMDAN-IWT and a rolling prediction scheme provides a ready-to-use solution for metro deep excavation monitoring. Since it is data-driven, applying it to a similar site only requires retraining the model on local data; the core denoising and prediction architecture remains unchanged.

In summary, this study developed a deformation prediction method for deep excavations based on the CEEMDAN-IWT joint denoising strategy and BO-XGBoost model. The method was validated through field data from the Wuhan metro deep excavation project, and a rolling prediction scheme was embedded to enable practical application. The developed strategy effectively improved monitoring data quality and reduced prediction errors, providing a practical tool with promising accuracy. Although the approach is data-driven and theoretically extendable, practical application requires retraining the model with local data and adjusting parameters.

## Figures and Tables

**Table 1:** Comparison of existing wavelet threshold selection methods.

Method Type	Continuity	Unbiasedness	Parameter Sensitivity	Main Limitations
Hard thresholding	No	Yes	Low	Discontinuous, Gibbs phenomenon
Soft thresholding	Yes	No	Low	Constant bias, signal energy compression
Semi-soft thresholding	Yes	No	Medium	Requires additional parameters, complex selection
Garrote thresholding	Yes	No	Medium	Large coefficients still have bias
Exponential thresholding	Yes	No	High	Sensitive to exponent base
Polynomial thresholding	Yes	Approximate	High	High computational cost
Logarithmic thresholding	Yes	No	Medium	Sensitive to logarithm base
Adaptive thresholding	Yes	Yes	Medium	Depends on prior knowledge
Proposed method	Yes	Yes	Low (asymptotic)	Depends on a tunable parameter

**Table 2:** Soil parameters.

Soil Layer	Unit Weight (kN/m^3^)	Elastic Modulus (MPa)	Poisson’s Ratio	Cohesion (kPa)	Internal Friction Angle (°)
Miscellaneous Fill	18.3	5.4	0.35	5	19
Plain Fill	18.2	7.0	0.34	9	7
Clay	18.8	12.0	0.25	18	11
Silty Sand	19.2	27.0	0.32	12	12
Pebble	20.0	115.0	0.29	0	35
Highly Weathered Silty Mudstone	23.4	215.0	0.26	110	25
Moderately Weathered Silty Mudstone	24.5	260.0	0.23	220	31

**Table 3:** Comparison of denoising evaluation indexes.

Signal	SNR/dB	RMSE/mm
signal containing noise	13.47	0.59
CEEMDAN-wavelet thresholding of jointly denoised signals	20.04	0.28
CEEMDAN-improved wavelet thresholding of jointly denoised signals	20.61	0.26

**Table 4:** Denoising results of exponential curve (k=−10).

Denoised Signal	Parameters b	42.5		85	
	Parameters δ	**0.4**	**0.6**	**0.4**	**0.6**
CEEMDAN-wavelet thresholding of jointly denoised signals	SNR/dB	21.93	19.76	27.49	25.58
	RMSE/mm	0.15	0.19	0.10	0.12
CEEMDAN-IWT of jointly denoised signals	SNR/dB	22.28	19.85	27.54	25.58
	RMSE/mm	0.14	0.19	0.10	0.12

**Table 5:** Denoising results of exponential curve (k=−15).

Denoised Signal	Parameters b	42.5		85	
	Parameters δ	0.4	0.6	0.4	0.6
CEEMDAN-wavelet thresholding of jointly denoised signals	SNR/dB	25.93	20.04	27.84	28.79
	RMSE/mm	0.14	0.28	0.14	0.13
CEEMDAN-IWT of jointly denoised signals	SNR/dB	26.05	20.61	28.10	28.79
	RMSE/mm	0.14	0.26	0.14	0.13

**Table 6:** Poisson curve denoising results (a=9, k=−15).

Denoised Signal	Parameters b	0.1		0.11	
	Parameters δ	0.4	0.6	0.4	0.6
CEEMDAN-wavelet thresholding of jointly denoised signals	SNR/dB	25.93	24.83	25.30	19.09
	RMSE/mm	0.12	0.14	0.12	0.25
CEEMDAN-IWT of jointly denoised signals	SNR/dB	26.04	24.83	25.36	19.87
	RMSE/mm	0.12	0.14	0.12	0.23

**Table 7:** Poisson curve denoising results (a=9, k=−20).

Denoised Signal	Parameters b	0.1		0.11	
	Parameters δ	0.4	0.6	0.4	0.6
CEEMDAN-wavelet thresholding of jointly denoised signals	SNR/dB	26.53	26.74	27.10	25.66
	RMSE/mm	0.15	0.15	0.13	0.16
CEEMDAN-IWT of jointly denoised signals	SNR/dB	26.75	26.75	27.28	25.69
	RMSE/mm	0.15	0.15	0.13	0.16

**Table 8:** Prediction results of the BO-XGBoost model based on raw monitoring data.

Phase	Measured Value/mm	Projected Value/mm	Absolute Error/mm	Relative Error/%
16	−2.27 −2.16	−2.08 −2.10	0.19 0.06	8.37 2.78
17	−2.04 −2.11	−2.28 −2.32	0.24 0.21	11.76 9.95
18	−2.18 −2.28	−2.35 −2.40	0.17 0.12	7.80 5.26
19	−2.37 −2.43	−2.45 −2.49	0.08 0.06	3.38 2.47
20	−2.48 −2.54	−2.52 −2.50	0.04 0.04	1.61 1.57

**Table 9:** Comparison of prediction error metrics between raw data and denoised data.

Data Source	RMSE (mm)	MAPE (%)	Maximum Absolute Error (mm)	Maximum Relative Error (%)
Raw Noisy Data	0.14	5.50	0.24	11.76
CEEMDAN-IWT Denoised Data	0.09	3.54	0.16	7.84
Improvement	35.7%	35.6%	33.3%	33.3%

**Table 10:** Construction of training and testing samples under the rolling prediction strategy.

Sample Type	Sample ID	Input Periods	Output Period	Data Source
Training	1	Periods 1–7	Period 8	Measured values (Training set)
Training	2	Periods 2–8	Period 9	Measured values (Training set)
Training	3	Periods 3–9	Period 10	Measured values (Training set)
Training	4	Periods 4–10	Period 11	Measured values (Training set)
Training	5	Periods 5–11	Period 12	Measured values (Training set)
Training	6	Periods 6–12	Period 13	Measured values (Training set)
Training	7	Periods 7–13	Period 14	Measured values (Training set)
Training	8	Periods 8–14	Period 15	Measured values (Training set)
Testing (Rolling Step 1)	1	Periods 9–15	Period 16	Input: measured; Output: predicted
Testing (Rolling Step 2)	2	Periods 10–15 + Predicted Period 16	Period 17	Input: measured + predicted; Output: predicted
Testing (Rolling Step 3)	3	Periods 11–15 + Predicted Periods 16–17	Period 18	Input: measured + predicted; Output: predicted
Testing (Rolling Step 4)	4	Periods 12–15 + Predicted Periods 16–18	Period 19	Input: measured + predicted; Output: predicted
Testing (Rolling Step 5)	5	Periods 13–15 + Predicted Periods 16–19	Period 20	Input: measured + predicted; Output: predicted

**Table 11:** Optimization range of hyperparameters to be optimized.

Hyperparameters to Be Optimized	Scope of the Search for Excellence
down sampling	[0.1, 1]
percentage of columns	[0.1, 1]
maximum depth of decision tree	[1, 10]
gamma function	[0.1, 0.2]

**Table 12:** Final hyperparameter configuration of the BO-XGBoost model.

Hyperparameter	Description	Search Range	Selected Value
n_estimators	Number of boosting rounds	[100, 1000]	450
learning_rate	Step size shrinkage	[0.01, 0.3]	0.08
max_depth	Maximum tree depth	[1, 10]	4
min_child_weight	Minimum sum of instance weight in a child	[1, 10]	3
subsample	Subsample ratio of training instances	[0.5, 1.0]	0.75
colsample_bytree	Subsample ratio of columns per tree	[0.1, 1.0]	0.65
reg_alpha	L1 regularization term	[0, 1]	0.10
reg_lambda	L2 regularization term	[0, 1]	0.85
gamma	Minimum loss reduction required for split	[0.1, 0.2]	0.12
booster	Type of booster	default	gbtree (fixed)

**Table 13:** BO-XGBoost model prediction results.

Phase	Measured Value/mm	Projected Value/mm	Absolute Error/mm	Relative Error/%
16	−2.27	−2.15	0.12	5.29%
17	−2.04	−2.20	0.16	7.84%
18	−2.18	−2.27	0.09	4.13%
19	−2.37	−2.39	0.02	0.84%
20	−2.48	−2.44	0.04	1.61%

**Table 14:** Linear regression model prediction results.

Phase	Measured Value/mm	Projected Value/mm	Absolute Error/mm	Relative Error/%
16	−2.27	−2.01	0.26	11.45
17	−2.04	−2.12	0.08	3.92
18	−2.18	−2.23	0.05	2.29
19	−2.37	−2.34	0.03	1.27
20	−2.48	−2.45	0.03	1.21

**Table 15:** Polynomial regression model prediction results.

Phase	Measured Value/mm	Projected Value/mm	Absolute Error/mm	Relative Error/%
16	−2.27	−2.02	0.25	11.01
17	−2.04	−2.10	0.06	2.94
18	−2.18	−2.21	0.03	1.38
19	−2.37	−2.35	0.02	0.84
20	−2.48	−2.46	0.02	0.81

**Table 16:** XGBoost model prediction results.

Phase	Measured Value/mm	Projected Value/mm	Absolute Error/mm	Relative Error/%
16	−2.27	−2.18	0.09	3.96%
17	−2.04	−2.27	0.23	11.27%
18	−2.18	−2.31	0.13	5.96%
19	−2.37	−2.43	0.06	2.53%
20	−2.48	−2.54	0.06	2.42%

**Table 17:** LSTM model prediction results.

Phase	Measured Value/mm	Projected Value/mm	Absolute Error/mm	Relative Error/%
16	−2.27	−2.13	0.14	6.17%
17	−2.04	−2.17	0.13	6.37%
18	−2.18	−2.19	0.01	0.46%
19	−2.37	−2.21	0.16	6.75%
20	−2.48	−2.22	0.26	10.48%

**Table 18:** BP model prediction results.

Phase	Measured Value/mm	Projected Value/mm	Absolute Error/mm	Relative Error/%
16	−2.27	−2.04	0.23	10.13%
17	−2.04	−2.03	0.01	0.49%
18	−2.18	−2.01	0.17	7.80%
19	−2.37	−1.96	0.41	17.30%
20	−2.48	−1.78	0.70	28.23%

**Table 19:** Different model prediction results.

Predictive Model	RMSE/mm	MAPE/%
BP model	0.38	12.79%
LSTM model	0.16	6.05%
XGBoost model	0.13	5.23%
BO-XGBoost model	0.09	3.54%
Linear regression model	0.125	4.03%
Polynomial regression model	0.114	3.40%
GRU model	0.12	4.61%
CNN-LSTM model	0.11	4.18%
TCN model	0.10	3.89%

**Table 20:** Correlation coefficients between input features and output settlement.

Feature	St-1	St-2	St-3	St-4	St-5	St-6	St-7
Correlation Coefficient	0.92	0.88	0.83	0.77	0.71	0.65	0.58

## Data Availability

The data presented in this study are available at the request from the corresponding author.

## Abbreviations

The following abbreviations are used in this manuscript:

BO	Bayesian Optimization
BO-XGBoost	Bayesian Optimization-based Extreme Gradient Boosting
RMSE	Root Mean Square Error
SNR	Signal-to-Noise Ratio
LSTM	Long Short-Term Memory
BP	Back Propagation Neural Network
VMD	Variational Mode Decomposition
CPO	Crested Porcupine Optimizer
IWT	Improved Wavelet Threshold
CEEMD	Complete Ensemble Empirical Mode Decomposition
SDCAL	Signal-Data dual-domain Collaborative Anti-interference and Localization
WOBSS	Blind Source Separation and Wavelet Optimization
PSO	Particle Swarm Optimization
